# Supplementary figures and images for: Evolution of secondary cell number and position in the Drosophila accessory gland
Source: PLoS One. 2023 Oct 25;18(10):e0278811. doi: 10.1371/journal.pone.0278811 (PMC10599531; doi:10.1371/journal.pone.0278811)

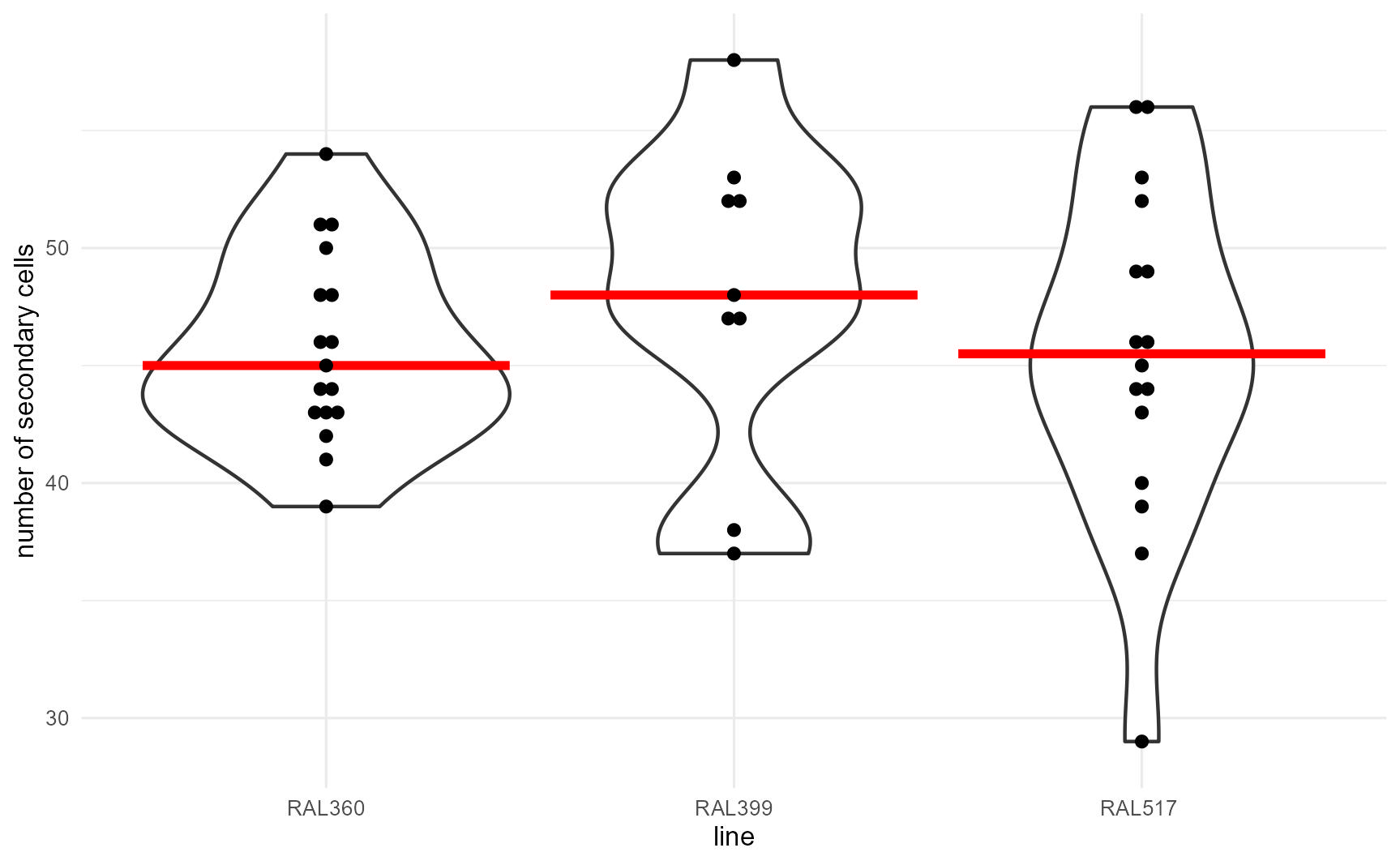

Supplement: S1 Fig — A summary of the secondary cell numbers within the 3 RAL lines examined (517, 360, 390). Red bars = mean. (TIF) [file pone.0278811.s001.tif]
